# A new giant keelback slug of the genus *Limax* from the Balkans, described by citizen scientists

**DOI:** 10.3897/BDJ.10.e69685

**Published:** 2022-06-20

**Authors:** Menno Schilthuizen, Cameron Graham Thompson, Rick de Vries, Anthonie D. P. van Peursen, Marta Paterno, Simone Maestri, Luca Marcolongo, Chiara Degli Esposti, Massimo Delledonne, Iva Njunjić

**Affiliations:** 1 Taxon Expeditions B.V., Leiden, Netherlands Taxon Expeditions B.V. Leiden Netherlands; 2 Naturalis Biodiversity Center, Leiden, Netherlands Naturalis Biodiversity Center Leiden Netherlands; 3 Department of Biological and Medical Sciences, Oxford Brookes University, Oxford, United Kingdom Department of Biological and Medical Sciences, Oxford Brookes University Oxford United Kingdom; 4 Amsterdam University of Applied Sciences, Amsterdam, Netherlands Amsterdam University of Applied Sciences Amsterdam Netherlands; 5 University of Verona, Verona, Italy University of Verona Verona Italy; 6 Department of Medicine, Surgery and Dentistry, University of Salerno, Baronissi, Italy Department of Medicine, Surgery and Dentistry, University of Salerno Baronissi Italy; 7 MNHN, Paris, France MNHN Paris France

**Keywords:** malacology, Limacidae, slugs, taxonomy, The Balkans, genitalia

## Abstract

**Background:**

Despite their large size, striking colouration and genital extravagance, the taxonomy of the European giant keelback slugs of the genus *Limax* is still poorly understood. Preliminary morphological and molecular data suggest that many unnamed or unrecognised species exist, especially in the Alps, the Mediterranean and the Balkans.

**New information:**

We organised a citizen science expedition to Durmitor National Park in Montenegro and discovered a new species, genetically distinct, but morphologically similar to the sympatric *L.cinereoniger* Wolf 1803 and describe it as *L.pseudocinereoniger*.

## Introduction

Given the long history and intensity of naturalist activity in central Europe, one would not expect there to remain any undiscovered animal species of 15-20 cm in body length. Yet, this is the case in the giant keelback slugs of the genus *Limax*. In general, these terrestrial animals (also known as 'tiger slugs' or 'leopard slugs') are well-known for their size and colour pattern (from cream via brick red to deep black, often with striking patterns of dots or bands). What also speaks to the imagination are their genitals (after barnacles, some *Limax* species have the longest penises in the animal kingdom, up to seven times their body length; Gerhardt 1933, Schilthuizen 2014) and mating behaviour (long, intricate 'dances' in which the hermaphroditic partners entwine their gigantic, extruded penises). Despite this familiarity, recent studies (Nitz et al. 2009, Nitz et al. 2010, Nitz 2013) have revealed that many European species remain unknown, unrecognised and/or unnamed.

The Balkan Peninsula is one of the regions where much unstudied and undescribed species-level *Limax* diversity exists. Nitz (2013) sequenced and morphologically studied a large number of individuals from this region and revealed at least 10 lineages, most referring to apparently undescribed endemic, short-range species. In fact, based on the fact that several Balkan lineages are early-branching, she suggests that the Balkans may be the region of origin of the entire genus.

In July 2018 and July 2019, we organised two citizen science expeditions to Durmitor National Park in Montenegro. These field trips were conceived by Taxon Expeditions (www.taxonexpeditions.com) to involve the general public in species discovery in biodiversity hotspots and to engage them in the description and publication of species new to science. Although early initiatives to involve citizen scientists in systematics studies did not move beyond the mere collecting of data (Silvertown 2009, Silvertown et al. 2011, Suprayitno et al. 2017), more recent projects take a much broader view and include a citizen science component in as large a part of the scientific process as possible, preferably from design until publication of a scientific study (Hecker et al. 2018, Haklay et al. 2021). Taxon Expeditions attempt to apply the latter, so-called "extreme citizen science" approach. Besides untrained citizen scientists, the teams consisted of professional taxonomists and ecologists, as well as ‘field geneticists’ who operate a mobile DNA lab allowing in-the-field sequencing of DNA barcode regions using Oxford Nanopore technology (Schilthuizen et al. 2017, Pomerantz et al. 2018, Maestri et al. 2019).

During the 2018 trip, we found and sequenced three large *Limax* specimens, which, based on Wiktor (1982), we initially assigned to the well-known, widespread *L.cinereoniger* Wolf, 1803. This species had been reported from the region earlier (Bole 1984). Upon comparison of the sequences with those in GenBank, however, it appeared that two distinct species were present. Two individuals indeed belonged to what is generally considered *L.cinereoniger*. The third individual, however, appeared conspecific with a seemingly undescribed sister species, informally named ‘*pseudocinereoniger*’ by Nitz (2013). Our Durmitor specimen had a COI sequence identical to ‘*pseudocinereoniger*’ from Biogradska Gora in Montenegro and nearly identical to samples of that taxon from Bulgaria. Here, we formally describe this form as a new species, genetically and (subtly) morphologically distinct from *L.cinereoniger*.

## Materials and methods

### Fieldwork

*Limax* specimens were collected by expedition members on two separate taxon expeditions to Durmitor National Park, Montenegro, 10-19 July 2018 and 9-18 July 2019. In addition, specimens were collected by the first author, M. Schilthuizen, in the summers of 2018 and 2019 in other locations in Montenegro. Specimens were retrieved from under logs and rocks on the forest floor, under overhanging rocks, in crevices in limestone and behind loose bark of dead trees. Individuals of mature size were photographed alive (where possible in dorsal, lateral and ventral view), relaxed overnight in water without air bubbles and then transferred into 70% ethanol. The ethanol was refreshed once or twice in the following days or weeks. Tissue samples of up to 10 mm^3^ were taken from the side of the sole or the keel and preserved in 96% ethanol for further molecular work. The details for specimens that were referred to *L.pseudocinereoniger* have been listed under "Types" in the description of that species below. The details for two specimens of *L.cinereoniger* (TxExDU0040-a and TxExDU0040-b in Fig. 4) are as follows: Montenegro, Durmitor National Park, Tara Canyon, 43.18136°N, 19.24084°E, 794 m elev., 13 July 2018, locality code: TxEx-DU0040, leg. I. Njunjić & Taxon Expedition participants, 2 adults (dissected and DNA-barcoded). All material is stored in the Taxon Expeditions Collection, Leiden (collection coden TXEX), Leiden, the collection of Naturalis Biodiversity Center, Leiden (collection coden RMNH) and the Zoologische Staatssammlung München (collection coden SNSB-ZSM).

### Morphology

For the descriptions of the morphology and the morphological differentiation of the new species from *L.cinereoniger*, we only used our own specimens of both species from Montenegro (see above under *Fieldwork*) and only those for which the species identity had been confirmed with a DNA barcode, i.e. the individuals marked with TxEx-DU locality codes in Fig. 4. The external appearance (colour patterns, sizes, proportions of body parts) was studied for adult specimens of the new species, based on photographs of the live animals and alcohol-preserved specimens before dissection. Preserved adults were dissected by first making an incision in the left flank immediately above the foot margin, then a crosswise incision just posterior of the eye tentacles and finally an incision around the genital opening. These three incisions and subsequent careful removal of connective tissue, allowed the separation of the genital system from the rest of the viscerum. The genital system was then pinned on a soft-bottom dissection dish and photographed with a scale bar in the image. For the anatomical terminology, we followed Nitz et al. (2009). However, the terminology used by Bodon et al. (2019), specifically for *L.cinereoniger*, deviates to some extent from the one of Nitz et al. (2009), as follows: "internal penial tongue" of Nitz et al. (2009) is the proximal ca. 40% of the "first crest" of Bodon et al. (2019); "longitudinal interior penial crest" of Nitz et al. (2009) is the distal ca. 60% of the "first crest" of Bodon et al. (2019); "second crest" of Bodon et al. (2019) is not separately identified by Nitz et al. (2009), but corresponds to the distalmost ca. 5-15% of the "longitudinal interior penial crest" of Nitz et al. (2009); the "longitudinal interior penial cord" of Nitz et al. (2009) corresponds to the "pleat" of Bodon et al. (2019).

### Molecular work

Tissue samples (prepared as described above) from eight individuals (see Supplementary Material) were analysed genetically with a portable field lab, as described previously (Menegon et al. 2017, Maestri et al. 2019). DNA was isolated with the DNeasy Blood & Tissue Kit (Qiagen) and then used in a PCR to amplify the COI barcoding region using the primers of Folmer et al. (1994). Sequencing was done on MinION and Flongle machines (Oxford Nanopore Technologies) as described in detail in Maestri et al. (2019). Reads were analysed with the ‘ONtoBAR’ and 'ONtrack' pipelines as described by Menegon et al. (2017) and Maestri et al. (2019). The sequences (and accompanying photos of live, preserved and dissected animals) were uploaded to the Barcoding of Life Database (BOLD; www.boldsystems.org). They are accessible under numbers TXEX039-19 – TXEX045-19 and TXEX048-19. To determine the species identities, we obtained all sequences of the cytochrome c oxidase subunit I (COI) barcoding region labelled in Nitz (2013) as ‘*Limaxcinereoniger*’, ‘*Limaxcf.cinereoniger*’ and ‘*Limax* “*pseudocinereoniger*”’ from GenBank. All sequences (newly generated as well as mined from GenBank) were aligned in Geneious Prime v.2020.0.4 using the MUSCLE algorithm (Edgar 2004) and default settings. The alignment (consisting of 31 sequences) was then subjected to a Maximum-Likelihood analysis using PhyML as incorporated in Geneious Prime (Guindon et al. 2010), with a GTR substitution model, fixed proportion of invariable sites, four substitution rate categories, estimated gamma distribution and 100 bootstrap replicates. Trees were displayed and manipulated in FigTree v.1.4.2 and *Lehmanniamarginata* (Genbank FJ606455) was used as outgroup to root the trees.

## Taxon treatments

### Limax pseudocinereoniger

Schilthuizen, Thompson, de Vries, van Peursen, Reisinger, Paterno, Maestri, Marcolungo, Esposti, Delledonne & Njunjić, 2022 sp. n.

F2336F99-5594-589F-B818-BE0F84DE63CF

3944596F-E524-4EED-97DB-FBA2A028687B

**Suborder Stylommatophora A. Schmidt, 1855**
**Superfamily Limacoidea Lamarck, 1801**
**Family Limacidae Lamarck, 1801**
**Genus *Limax* Linnaeus, 1758**
Type species: *Limaxmaximus* Linnaeus, 1758
synonyms:
Limax “*pseudocinereoniger*” Nitz (2013)

#### Materials

- **Type status:** Holotype. **Occurrence:** occurrenceDetails: http://www.boldsystems.org/index.php/API_Public/specimen?ids=TxExDU0122|TxExPR0004|TxExDU0041|TxExDU0112|TxExDU0121; recordNumber: TxExDU0041; recordedBy: Taxon Expeditions participants; individualID: TxExDU0041; individualCount: 1; sex: H; associatedMedia: http://www.boldsystems.org/pics/TXEX/slug_3+1577602544.jpg|http://www.boldsystems.org/pics/TXEX/Giant_slug+1577607140.JPG|http://www.boldsystems.org/pics/TXEX/slug_3_lateral+1577604939.jpg|http://www.boldsystems.org/pics/TXEX/slug_3_ventral+1577605013.jpg|http://www.boldsystems.org/pics/TXEX/slug_3_dorsal+1577604893.jpg; **Taxon:** scientificName: *Limaxpseudocinereoniger*; phylum: Mollusca; class: Gastropoda; order: Stylommatophora; family: Limacidae; genus: Limax; specificEpithet: *pseudocinereoniger*; **Location:** country: Montenegro; decimalLatitude: 43.2189; decimalLongitude: 19.1759; **Event:** eventDate: 18-07-2018; habitat: under rocky overhang; **Record Level:** institutionCode: Taxon Expeditions B.V.; basisOfRecord: PreservedSpecimen
- **Type status:** Paratype. **Occurrence:** occurrenceDetails: http://www.boldsystems.org/index.php/API_Public/specimen?ids=TxExDU0122|TxExPR0004|TxExDU0041|TxExDU0112|TxExDU0121; recordNumber: TxExDU0122; recordedBy: Menno Schilthuizen; individualID: TxExDU0122; individualCount: 1; sex: H; associatedMedia: http://www.boldsystems.org/pics/TXEX/slug_4+1577602643.jpg|http://www.boldsystems.org/pics/TXEX/DU0122lateral+1577615238.jpg|http://www.boldsystems.org/pics/TXEX/DU0122ventral+1577615289.jpg|http://www.boldsystems.org/pics/TXEX/slug_4_dorsal+1577605110.jpg|http://www.boldsystems.org/pics/TXEX/DU0122dorsal+1577615162.jpg|http://www.boldsystems.org/pics/TXEX/slug_4_lateral+1577605161.jpg|http://www.boldsystems.org/pics/TXEX/slug_4_ventral+1577605254.jpg; **Taxon:** scientificName: *Limaxpseudocinereoniger*; phylum: Mollusca; class: Gastropoda; order: Stylommatophora; family: Limacidae; genus: Limax; specificEpithet: *pseudocinereoniger*; **Location:** country: Montenegro; decimalLatitude: 43.169; decimalLongitude: 18.999; **Identification:** identifiedBy: Menno Schilthuizen; **Record Level:** institutionCode: Taxon Expeditions B.V.; basisOfRecord: PreservedSpecimen
- **Type status:** Paratype. **Occurrence:** occurrenceDetails: http://www.boldsystems.org/index.php/API_Public/specimen?ids=TxExDU0122|TxExPR0004|TxExDU0041|TxExDU0112|TxExDU0121; recordNumber: TxExDU0112; recordedBy: M. Schilthuizen & I. Njunjić; individualID: TxExDU0112; individualCount: 1; sex: H; associatedMedia: http://www.boldsystems.org/pics/TXEX/TxEx-DU0112-Lim-dorsal+1577602220.jpg|http://www.boldsystems.org/pics/TXEX/slug_5_lateral+1577605397.jpg|http://www.boldsystems.org/pics/TXEX/slug_5_dorsal+1577605353.jpg|http://www.boldsystems.org/pics/TXEX/slug_5+1577602761.jpg|http://www.boldsystems.org/pics/TXEX/TxEx-DU0112-Lim-ventral+1577602257.jpg|http://www.boldsystems.org/pics/TXEX/slug_5_ventral+1577605440.jpg; **Taxon:** scientificName: *Limaxpseudocinereoniger*; phylum: Mollusca; class: Gastropoda; order: Stylommatophora; family: Limacidae; genus: Limax; specificEpithet: *pseudocinereoniger*; **Location:** country: Montenegro; locality: Arapova Pećina; decimalLatitude: 43.0481; decimalLongitude: 19.0793; **Identification:** identifiedBy: Menno Schilthuizen; **Record Level:** institutionCode: Taxon Expeditions B.V.; basisOfRecord: PreservedSpecimen
- **Type status:** Paratype. **Occurrence:** occurrenceDetails: http://www.boldsystems.org/index.php/API_Public/specimen?ids=TxExDU0122|TxExPR0004|TxExDU0041|TxExDU0112|TxExDU0121; recordNumber: TxExDu0121; recordedBy: Rick de Vries; individualID: TxExDU0121; individualCount: 1; sex: H; associatedMedia: http://www.boldsystems.org/pics/TXEX/slug_7+1577602879.jpg|http://www.boldsystems.org/pics/TXEX/slug_7_lateral+1577605850.jpg|http://www.boldsystems.org/pics/TXEX/DU0121ventral+1577614992.jpg|http://www.boldsystems.org/pics/TXEX/slug_7_dorsal+1577605804.jpg|http://www.boldsystems.org/pics/TXEX/DU0121dorsal+1577614812.jpg|http://www.boldsystems.org/pics/TXEX/DU0121lateral+1577614952.jpg|http://www.boldsystems.org/pics/TXEX/slug_7_ventral+1577605892.jpg; **Taxon:** scientificName: *Limaxpseudocinereoniger*; phylum: Mollusca; class: Gastropoda; order: Stylommatophora; family: Limacidae; genus: Limax; specificEpithet: *pseudocinereoniger*; **Location:** country: Montenegro; decimalLatitude: 43.2189; decimalLongitude: 19.1759; **Identification:** identifiedBy: Menno Schilthuizen; **Record Level:** institutionCode: Taxon Expeditions B.V.; basisOfRecord: PreservedSpecimen
- **Type status:** Paratype. **Occurrence:** occurrenceDetails: http://www.boldsystems.org/index.php/API_Public/specimen?ids=TxExDU0122|TxExPR0004|TxExDU0041|TxExDU0112|TxExDU0121; recordNumber: TxExPr0004; recordedBy: M. Schilthuizen & I. Njunjić; individualID: TxExPR0004; individualCount: 1; sex: H; associatedMedia: http://www.boldsystems.org/pics/TXEX/TxExPR0004vent+1577624611.JPG|http://www.boldsystems.org/pics/TXEX/TxExPR0004dors+1577624546.JPG|http://www.boldsystems.org/pics/TXEX/TxExPR0004late+1577624578.JPG; **Taxon:** scientificName: *Limaxpseudocinereoniger*; phylum: Mollusca; class: Gastropoda; order: Stylommatophora; family: Limacidae; genus: Limax; specificEpithet: *pseudocinereoniger*; **Location:** country: Montenegro; locality: Katun Zastan; decimalLatitude: 42.5203; decimalLongitude: 19.7855; **Identification:** identifiedBy: Menno Schilthuizen; **Record Level:** institutionCode: Taxon Expeditions B.V.; basisOfRecord: PreservedSpecimen

#### Description

Holotype: Montenegro, Durmitor National Park, Tara Canyon, 43.21888°N, 19.17588°E, 783 m elev., under rocky overhang, 13 July 2018, locality code: TxEx-DU0041, leg. I. Njunjić & Taxon Expedition participants, 1 adult (dissected and DNA-barcoded): RMNH.MOL.338775 in RMNH, Naturalis Biodiversity Center, Leiden, the Netherlands.

Paratypes: Montenegro, Durmitor National Park, Sušićko Valley, 43.16900°N; 18.99900°E, 1210 m elev., behind loose bark of log, 13 July 2019, locality code: TxEx-DU0122, leg. M. Schilthuizen & Taxon Expedition participants, 1 adult (dissected and DNA-barcoded) in TXEX, Leiden, The Netherlands. Montenegro, Grabovica, 43.04809°N; 19.07934°E, 1564 m elev., under log, 5 July 2019, locality code: TxEx-DU0112, leg. M. Schilthuizen & I. Njunjić, 1 adult (dissected and DNA-barcoded) in TXEX, Leiden, The Netherlands. Montenegro, Durmitor National Park, Tara Canyon, 43.21888°N, 19.17588°E, 783 m elev., in crevice in rock, 12 July 201), locality code: TxEx-DU0121, leg. R de Vries & Taxon Expedition participants, 1 adult (dissected and DNA-barcoded) in TXEX, Leiden, The Netherlands. Montenegro, Prokletije, Katun Zastan, 42.52031°N, 19.78551°E, 1296 m elev., 24 July 2019, leg. M. Schilthuizen & I. Njunjić, 1 adult (DNA-barcoded) in SNSB-ZSM.

**Other material.** Montenegro, Biogradska Gora (BNM 060820); Bulgaria, Vitosha-Rila-Rhodopes (BNM 062850, BNM 060529, BNM 060561, BNM 063021). (Codes refer to the collection of the Bündner Naturmuseum Chur; material was used for sequencing by B. Nitz, but not studied morphologically by us.)

**External appearance.**

Large, up to 116 mm long; mantle length up to 37 mm; keel length up to 44 mm (measurements based on alcohol-preserved specimens; living animals can be considerably larger when fully extended). Keel prominent. Colouration monochrome or patterned. Body colour dark brown to black, fading to light brown on the flanks or uniformly light brown (preserved specimens light grey, fading to creamy white on the flanks or uniformly grey to black). Dorsum often darker than the flanks. Keel often (but not always) distinctly brighter than the rest of the dorsal body colour. Mantle colour similar to or darker than the dorsum, always without any patterning (preserved specimens have the mantle similar or lighter than the rest of the dorsum). Inner field of the tripartite sole of the foot always creamy white, outer fields mottled grey or black, fading from posterior to anterior and from the outer edge towards the inner field (similar colouration in preserved specimens). Head colour similar to or lighter than the body, darker dorsally than laterally, sometimes with spotted pattern around the mouth area and on the tentacles. Eye tentacles dark grey to black or creamy white with dark pigmented spots (similar colouration in preserved specimens).

**Genitalia.** (Based on dissections of five individuals from Montenegro.) See Figs 2, 3. Hermaphrodite duct long, distally thicker and coiled, cream in colour. Albumen gland yellowish, finger-shaped. Spermoviduct folded. Oviduct white. Free oviduct with capsular gland well developed. Vagina absent. Duct of bursa copulatrix inserts into free oviduct very near to junction with the penis; duct and sac not clearly distinct, sac oval or pear-shaped, fixed with connecting fibres to free oviduct. Atrium very short, almost invisible. Penis tubular, of nearly uniform thickness (but with a bulge of thickened penis wall roughly in the middle), 78 – 90 mm in adult animals or about four-fifths length of body in preserved stage, distal part with ca. four zigzag-bends; proximal part straight, but the final 10-15 mm closest to the vas deferens bent under a sharp angle. Vas deferens inserted close to penis end, leaving 1 – 3 mm blind round tip; penis retractor muscle attached to penis immediately proximal from vas deferens; vas deferens enters penis with a short invaginated papilla. In the penis interior, a short, inconspicuous longitudinal interior penial cord is present only in the distal one-fifth (or less) of the penis. The transversely striated longitudinal interior penial crest runs between (distally) the opening of the duct of the bursa copulatrix and continuing to (proximally) the level of the thickened penis wall. Slightly distal from this point, a more prominent, transversely striated fan-like structure (the internal penial tongue) is developed, which proximally rises to a height similar to the circumference of the penis. In some individuals, the internal penial tongue is contiguous with the longitudinal interior penial crest, but in others, they are separate and run parallel for a short distance. Longitudinal interior penial crest with very fine papillae at the root and towards the proximal part structured with numerous very fine transverse chamfers. Distal half of penis wall internally covered with fine, weak transverse riblets; proximal portion of penis wall smooth without any visible accessory structures besides entrance of vas deferens.

**Copulation.**

Mating behaviour is important for species distinction in *Limax* (Nitz et al. 2009, Nitz 2013). Like *L.cinereoniger* and, based on Fig. 8.5 and Table 8.2 in Nitz (2013) for a copula from Rila, Bulgaria, mating couples of *L.pseudocinereoniger* do not suspend themselves from a mucus thread (as is the case in most other *Limax* species), but instead hang from a mucus spot or mucus 'sail'.

**DNA barcode.**

The COI barcode of the holotype specimen (BOLD registration code TXEX041-19) is given below. Due to the low quality, we trimmed the 5' and 3' ends by 33 and 50 nucleotides, respectively. However, full DNA barcodes are available in BOLD for the paratypes.

5'TATAGTAGGAACAGGTTTATCTTTATTAATTCGGTTAGAGTTGGGAACAGCGGGCGTTTTAATAGATGATCACTTTT TTAATGTGATTGTAACTGCTCATGCATTTGTTATAATTTTTTTTATAGTAATACCAATTATGATTGGAGGTTTTGGTAATT GAATGGTTCCACTATTAATTGGAGCTCCCGATATAAGATTTCCTCGAATAAACAATATAAGGTTTTGATTATTACCACCT TCTTTTATTTTACTTATTTGTTCTAGTATGGTAGAGGGTGGTGCAGGTACAGGGTGAACTGTATATCCACCTTTAAGGG GACCTTTAGGTCATGGGGGAGCTTCTGTAGATTTAGCTATTTTTTCATTGCATTTAGCTGGGATGTCTTCTATTTTAGG GGCTATTAATTTTATTACAACTATTTTTAACATACGAACGTCAGGGATAACTATAGAACGTGTGAGGTTATTTGTTTGG TCTATTTTAGTAACTGTTTTTCTACTTTTGTTATCTCTTCCTGTATTAGCAGGGGCAATTACTATACTTTTAACAGATCG TAATTTTAATACTAGGT3'

#### Diagnosis

**Figure 1.**
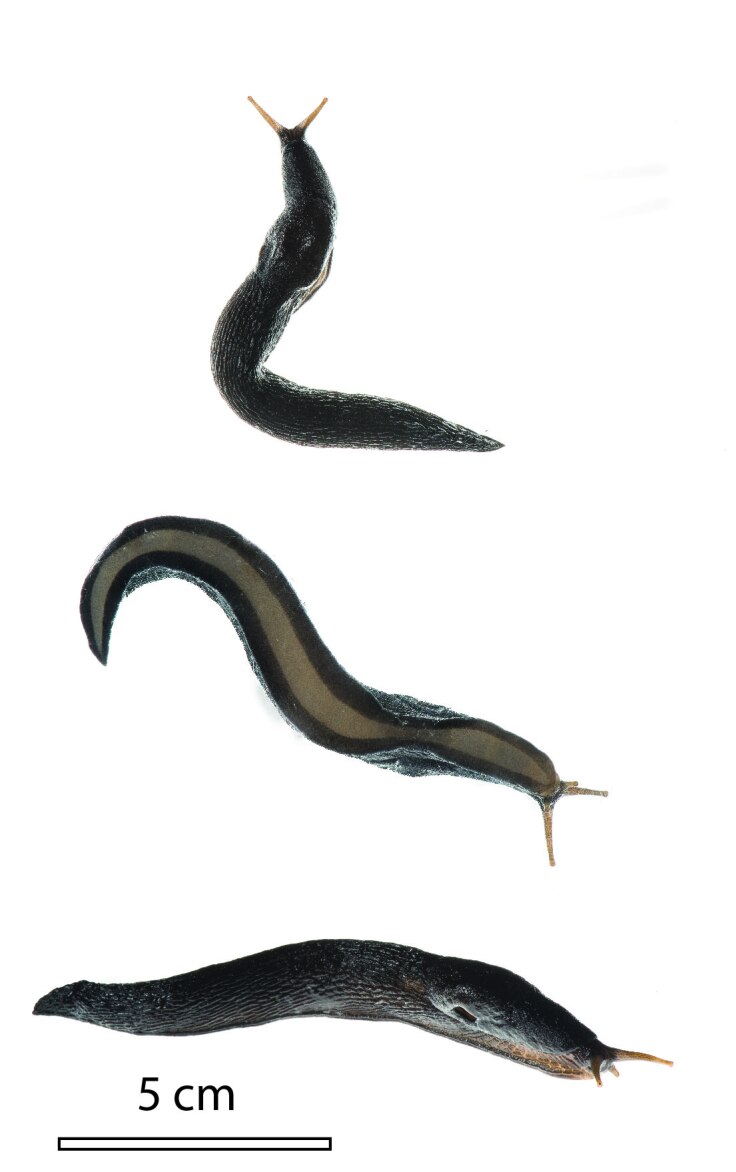
Dorsal (top), lateral (centre) and ventral (bottom) views of a living (and subsequently preserved) paratype specimen of *L.pseudocinereoniger* from Durmitor National Park, Montenegro (TxEx-DU0122). Photos by P. Escoubas.

**Figure 2.**
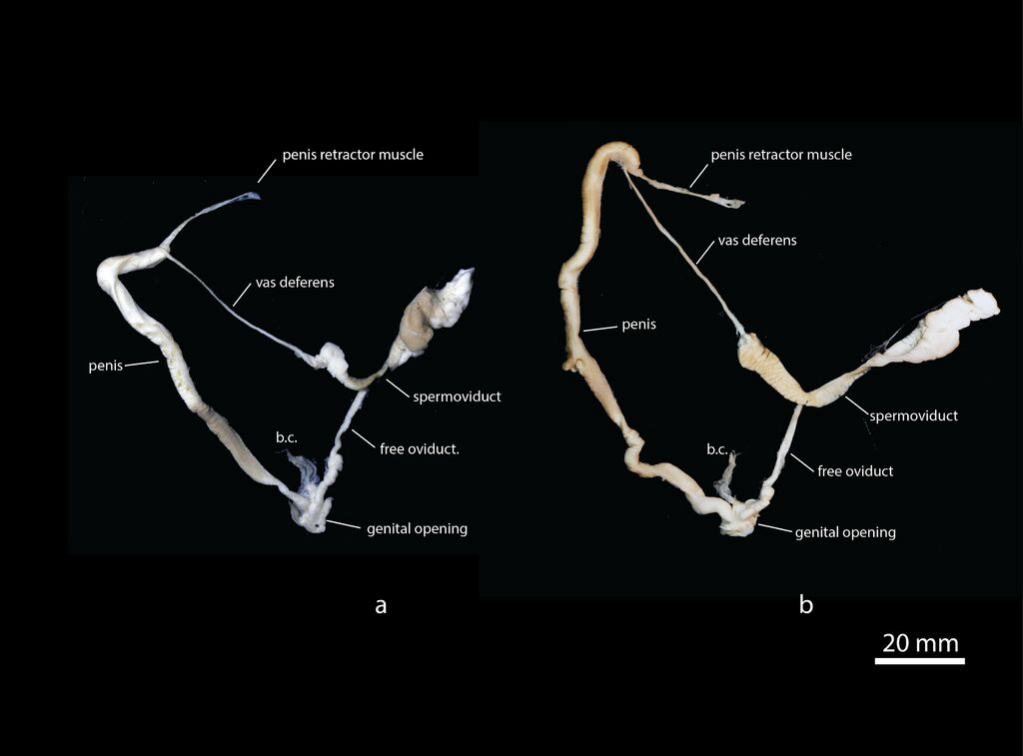
Genital dissections of sympatric *Limaxpseudocinereoniger* n. sp. (a; holotype, TxExDU0041) and *L.cinereoniger* (b; TxExDU0040) from Tara Canyon, Durmitor National Park, Montenegro (b.c. = bursa copulatrix).

**Figure 3.**
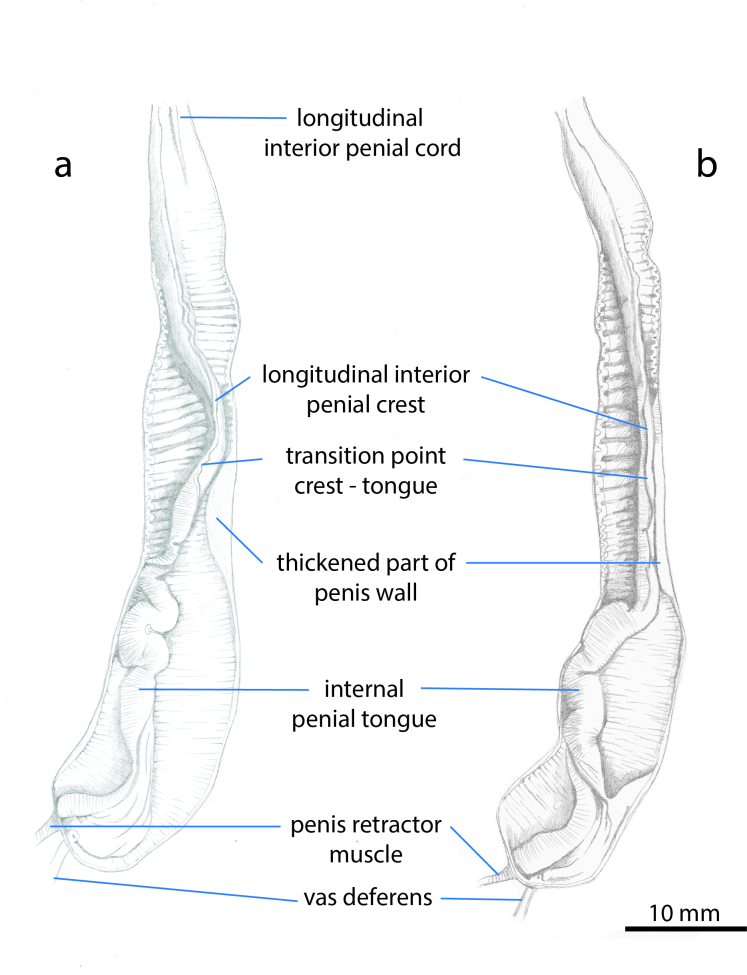
Interior structure of the penis of: (a) *L.pseudocinereoniger* from Tara Canyon, Montenegro (TxEx-DU0041) and (b) an individual of *L.cinereoniger* from Tara Canyon. Montenegro (TxEx-DU0040).

In external appearance (size and colouration, Fig. 1), *L.pseudocinereoniger* is very similar to *L.cinereoniger*, also in its variability in colour and colour pattern. Based on our own observations, as well as those in Nitz (2013) and Bodon et al. (2019), the dorsum is more often brownish than in *L.cinereoniger*, but otherwise no consistent external distinguishing marks could be discovered. Besides the nearly 10% differentiation in COI-sequence, a few subtle marks of distinction between the two taxa were found in the genitalia. We have listed these below, with indications on whether the distinguishing characters may be generally or only locally applicable.

(i) the one-sided bulge of thickened penis wall of *L.pseudocinereoniger* is absent in the sympatric *L.cinereoniger* and we also do not see any evidence of it in the images of the genitalia of *L.cinereoniger* in Bodon et al. (2019).

(ii) the short longitudinal interior penial cord is distinct, whereas in the sympatric Montenegrin *L.cinereoniger* specimens that we studied, it is absent or very inconspicuous. However, Bodon et al. (2019) depict animals of *L.cinereoniger* from other localities in which the longitudinal interior penial cord is as well-developed as in *L.pseudocinereoniger*. It may, therefore, well be a distinction that only applies to the Montenegrin region of sympatry.

(iii) the longitudinal interior penial crest in *L.pseudocinereoniger* is highest in its proximal half, whereas in the sympatric Montenegrin *L.cinereoniger*, it is highest in its distal half. We cannot observe this character in the dissections published by Bodon et al. (2019) for various parts of the *L.cinereoniger* range, so it may well be a distinction that only applies to the Montenegrin region of sympatry.

#### Etymology

The specific epithet *pseudocinereoniger* refers to its similarity with *L.cinereoniger*. This name was first applied as a "working name" by Nitz (2013) and is here adopted as the formal name. It is used as a masculine adjective.

The taxonomic authority for this species is attributed to all authors of this publication. In line with ICZN Recommendation 51C (Zoological Nomenclature 1999), the species may be referred to as *Limaxpseudocinereoniger* Schilthuizen et al., 2022, provided the full citation of this publication appears in the bibliography or elsewhere in the referring work.

## Discussion

**Figure 4.**
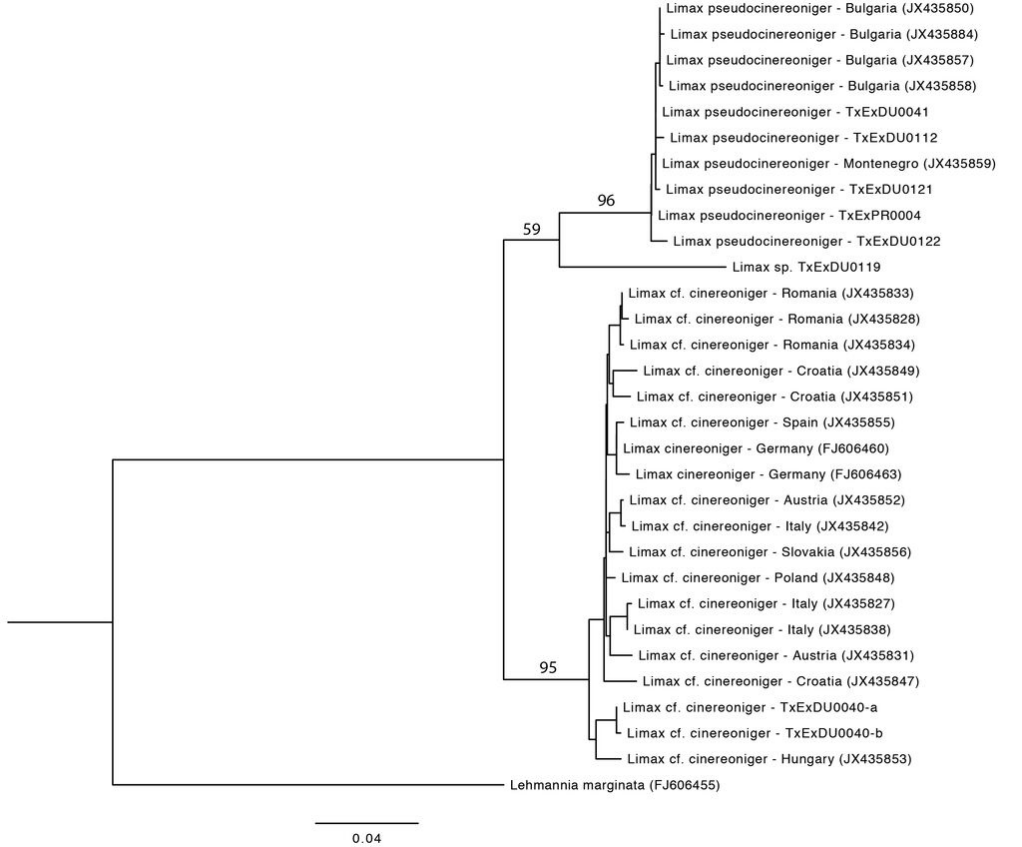
Phylogenetic (PhyML) reconstruction of *L.cinereoniger*, *L.pseudocinereoniger* and the unassigned TxExDU0119, based on DNA-barcode COI sequences and rooted with *Lehmanniamarginata*. Bootstrap percentages have been indicated for the major groupings.

Our phylogenetic analysis (Fig. 4) reveals that all sequences from specimens identified in Nitz et al. (2010), either as *L.cinereoniger* or *L.cf.cinereoniger* (deriving from a large area throughout central and southern Europe), as well as two specimens from the Tara Canyon (TxExDU0040) in Durmitor National Park, indeed form a relatively cohesive molecular clade which appears to represent a single species, *L.cinereoniger*. All specimens from Bulgaria and Montenegro identified in Nitz et al. (2010) as *L. 'pseudocinereoniger*', together with five specimens collected in Durmitor National Park and in the Prokletije Mountains in Montenegro (TxExDU0041, TxExDU0112, TxExDU0121, TxExDU0122 and TxExPR0004) form a different, genetically uniform clade with a genetic distance of ca. 10% from *L.cinereoniger*. Since this degree of divergence is similar to the differentiation seen amongst other species in *Limax* (e.g. *L.maximus* vs. *L.brandstetteri* (Nitz 2013) and since *L.cinereoniger* and *L.pseudocinereoniger* are sympatric at least in the Durmitor region, we feel justified in recognising it as a separate species, even though the morphological differentiation is only slight. A single individual, from locality TxExDU0119 in the Sinjajevina Heights, east of Durmitor, takes a genetically differentiated position in between *L.cinereoniger* and *L.pseudocinereoniger* and is probably a third, yet undescribed species, which we hope to return to in a future study. We have not further studied it morphologically for the present paper. However, based on its molecular phylogenetic position, as well as external colour patterns, it appears not to be conspecific with any of the other clades mentioned in Nitz (2013) (i.e. Clade 26, *Limax* sp. from Mt. Zekora Glava in Eastern Montenegro; Clade 27, *Limax* sp. from Albania; Clade 28, *Limax* "*pseudomaximus*" from Bulgaria).

L.cinereonigervar.schulzei Gerhardt, 1941, from the Rila Mountain Range in Bulgaria should be considered a species inquirenda. This taxon, which Gerhardt considered to differ in colouration (brown, not grey or black), anatomy (shorter caecum) and mating behaviour (copulation shorter and earlier in the day) was synonymised with *L.cinereoniger* *s. str.* by Wiktor (1982) and the name *schulzei* has not been in use since. The type specimens must be considered lost (personal communication, Christine Zorn, collection assistant of Mollusca, Museum für Naturkunde Berlin). However, given the description and the type locality, it is conceivable that the material used by Gerhardt to describe his var. schulzei is conspecific with *L.pseudocinereoniger*.

Based on the morphological data in Nitz (2013), *L.pseudocinereoniger* differs from Clades 26, 27 and 28 (Nitz 2013), three related undescribed species from the Balkan, in the following manner:

Clade 26 has a penis that is 1.2 times as long as the body length (0.8 times in *L.pseudocinereoniger*), brighter colour between the body's external wrinkles (uniform dark grey in *L.pseudocinereoniger*) and a short, inconspicuous keel (*L.pseudocinereoniger* has a long, pale keel).

Clade 27 has a penis that is 0.5 times as long as the body length (0.8 times in *L.pseudocinereoniger*), a penis that is nearly straight (coiled and folded in *L.pseudocinereoniger*), a completely pale sole (outer fields dark grey in *L.pseudocinereoniger*) and a short, inconspicuous keel (*L.pseudocinereoniger* has a long, pale keel).

Clade 28 has a mantle colour pattern consisting of some black dots and many white dots and a body pattern of two longitudinal rows of black dots, whereas *L.pseudocinereoniger* has a uniform dark grey body without any patterns of dots, a completely pale sole (outer fields dark grey in *L.pseudocinereoniger*) and a short, inconspicuous keel (*L.pseudocinereoniger* has a long, pale keel).

At least in the part of Montenegro that we studied, *L.pseudocinereoniger* appears to occur sympatrically with *L.cinereoniger*: the locations TxExDU0040 (*L.cinereoniger*) and TxExDU0041 (*L.pseudocinereoniger*), both in the bottom of the Tara Canyon, are located less than 7 km apart.

## Supplementary Material

XML Treatment for Limax pseudocinereoniger
